# An MDM2 antagonist (MI-319) restores p53 functions and increases the life span of orally treated follicular lymphoma bearing animals

**DOI:** 10.1186/1476-4598-8-115

**Published:** 2009-12-03

**Authors:** Ramzi M Mohammad, Jack Wu, Asfar S Azmi, Amro Aboukameel, Angela Sosin, Sherwin Wu, Dajun Yang, Shaomeng Wang, Ayad M Al-Katib

**Affiliations:** 1 Division of Hematology and Oncology, Department of Internal Medicine, Karmanos Cancer Institute, Wayne State University School of Medicine, 732 HWCRC, 4100 John R Street, Detroit, Michigan 48201, USA; 2 Detroit Country Day School, Beverly Hills, Michigan, Michigan, USA; 3 Department of Internal Medicine, University of Michigan, USA; 4 Ascenta Therapeutics, Inc, Malvern, Pennsylvania, USA

## Abstract

**Background:**

MI-319 is a synthetic small molecule designed to target the MDM2-P53 interaction. It is closely related to MDM2 antagonists MI-219 and Nutlin-3 in terms of the expected working mechanisms. The purpose of this study was to evaluate anti-lymphoma activity of MI-319 in WSU-FSCCL, a B-cell follicular lymphoma line. For comparison purpose, MI-319, MI-219 and Nutlin-3 were assessed side by side against FSCCL and three other B-cell hematological tumor cell lines in growth inhibition and gene expression profiling experiments.

**Results:**

MI-319 was shown to bind to MDM2 protein with an affinity slightly higher than that of MI-219 and Nutlin-3. Nevertheless, cell growth inhibition and gene expression profiling experiments revealed that the three compounds have quite similar potency against the tumor cell lines tested in this study. *In vitro*, MI-319 exhibited the strongest anti-proliferation activity against FSCCL and four patient cells, which all have wild-type p53. Data obtained from Western blotting, cell cycle and apoptosis analysis experiments indicated that FSCCL exhibited strong cell cycle arrest and significant apoptotic cell death; cells with mutant p53 did not show significant apoptotic cell death with drug concentrations up to 10 μM, but displayed weaker and differential cell cycle responses. In our systemic mouse model for FSCCL, MI-319 was tolerated well by the animals, displayed effectiveness against FSCCL-lymphoma cells in blood, brain and bone marrow, and achieved significant therapeutic impact (p < 0.0001) by conferring the treatment group a > 28% (%ILS, 14.4 days) increase in median survival days.

**Conclusion:**

Overall, MI-319 probably has an anti-lymphoma potency equal to that of MI-219 and Nutlin-3. It is a potent agent against FSCCL *in vitro *and *in vivo *and holds the promises to be developed further for the treatment of follicular lymphoma that retains wild-type p53.

## Background

Follicular lymphoma is a slow growing B-cell lymphoma and is the second most common type of non-Hodgkin's lymphoma (NHL), which is expected to have more than 66,000 new cases in the USA in 2008 [1]. Despite improvement of survival rates in recent years [2,3], follicular lymphoma remains incurable due mainly to limitations of the current first-line standard of treatment, which usually involves concomitant administration of humanized anti-CD20 monoclonal antibody rituximab and a chemotherapy regimen [4]. In the pivotal clinical trial that led to the approval of rituximab for clinical use in the USA, only 48% of patients with relapsed follicular lymphoma responded [5]. Therefore, better therapeutics is needed to further improve the outcome of afflicted patients.

A growing number of recent reports suggest that small molecule inhibitors targeting the MDM2-p53 interaction may represent very promising, specific and novel therapeutics against various types of cancers [6-9]. The p53 gene is an important tumor suppressor. It can promote cell cycle arrest by up-regulating the expression of genes involved in cell cycle control, such as p21^WAF1 ^[10,11]; and can also promote apoptosis, possibly by the up-regulation of pro-apoptotic genes, such as Bax and PUMA [12-14]. Among all the cancer patients, approximately half of them have mutated or deleted p53 gene, which leads to defective p53 protein or complete missing of functional p53 protein [15,16]. Among the remaining patients with wild-type p53 gene, functional p53 protein is quickly degraded after protein translation, primarily through direct interaction with the MDM2 protein [17]. Thus, using small molecules to block the MDM2-p53 interaction is an attractive approach to stabilize functional p53 protein and restore its anti-tumor activity in tumors with wild-type p53 gene.

Unlike in many solid tumors, alterations of the p53 gene are far less common in hematological malignancies (generally < 15%) [18]. Therefore, small-molecule inhibitors that interrupt the MDM2-p53 interaction might represent a new therapeutic strategy for the treatment of most patients with this kind of disease. Previous studies demonstrated that a different inhibitor of MDM2, Nutlin-3, is indeed able to efficiently induce apoptosis in B-cell chronic lymphocytic leukemia (B-CLL) [19-24]. To our knowledge, however, there are no reports so far on the studies of this kind of small-molecule inhibitors against follicular lymphoma. In the present study, we report on the evaluation of a new inhibitor of the MDM2-p53 interaction, named MI-319, against a follicular small cleaved B-cell lymphoma line (FSCCL) [25]*in vitro *by using cultured cells and *in vivo *by using a systemic model in mice with severe combined immunodeficiency (SCID). MI-319 is closely related to MDM2 antagonists MI-219 [8] and Nutlin-3 [6] in terms of the expected main working mechanisms. For comparison purpose, we also assessed these three compounds side by side against FSCCL and three other B-cell hematological tumor cell lines in growth inhibition and gene expression profiling experiments.

## Results

### MI-319 binds to MDM2 protein with high affinity

**Figure 1:**
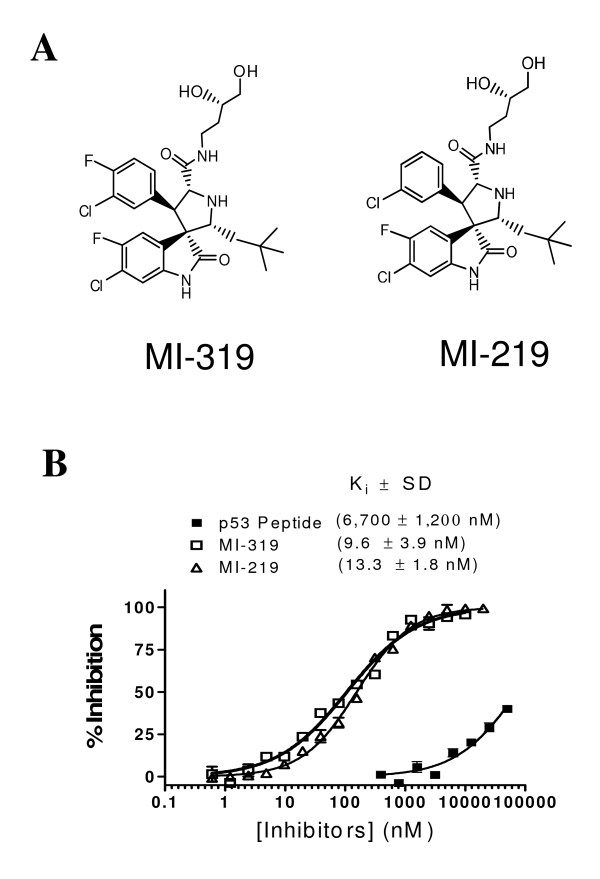
**Chemical structure of MI-319 and MI-219 and MDM2 protein binding assay**. (A), Chemical structure of MI-319 and MI-219. (B), Fluorescence polarization-based MDM2 binding assay. The binding affinities (*Ki *values) were determined by a competitive fluorescence polarization-based binding assay using recombinant His-tagged MDM2 (amino acids 1-118) and PMDM6-F (5-FAM-βAla-βAla-Phe-Met-Aib-pTyr-(6-Cl-_L_-Trp)-Glu-Ac3c-Leu-Asn-NH2), a fluorescently labeled high-affinity p53-based peptide.

MI-319 has a chemical structure very similar to that of MI-219 (Fig. 1A). The fluorescence polarization-based competitive binding assay determined that MI-319 binds to recombinant human MDM2 protein with a *K*_i _value of 9.6 ± 3.9 nmol/L, which is lower than the *K*_i _values of 13.3 ± 1.8 nmol/L and 36.0 ± 9.0 nmol/L determined for MI-219 (Fig. 1B) and Nutlin-3 [8], respectively. Therefore, MI-319 binds to human MDM2 protein with an affinity slightly higher than that of MI-219 and Nutlin-3. When compared with p53 protein - a natural MDM2 binding target, it appeared that both MI-319 and MI-219 were over 500 times more potent than a natural p53 peptide in binding to MDM2 under the same assay conditions (Fig. 1B).

### FSCCL cell growth can be effectively inhibited by MI-319, MI-219 and Nutlin-3

Because MDM2 antagonists such as MI-319 were expected to work mainly by restoring the activities of functional p53 protein, DNA sequencing was carried out first to determine the mutation status of *p53 *in the cells studied. *P53 *full-length coding cDNA sequences were sequenced for the four B-cell lines (WSU-FSCCL, WSU-WM, RL and WSU-DLCL_2_), whereas *p53 *genomic DNA sequences covering exons 5-9 were sequenced for four patient cells. Sequencing results are summarized in Table 1. We have used these four cell lines since they represent a wide spectrum of b-cell lineage tumors: (a) WSU-FSCCL [representing follicular low grade non-Hodgkin's lymphoma type that is wt-p53]; (b) WSU-WM [representing plasmacytoid type that is mut-p53]; (c) RL representing diffuse large B-cell lymphoma, mut-p53; (d) WSU-DLCL2 [representing diffuse, Intermediate grade non-Hodgkin's lymphoma mut-p53].

**Table 1:** Summary of cell P53 status

Cell	p53 mutation status
FSCCL	wild-type
WM	R213Q
RL	A138P
DLCL2	A248Q
BP071708	wild-type
RM072307	wild-type
CH012306	wild-type
JC012706	wild-type

**Figure 2:**
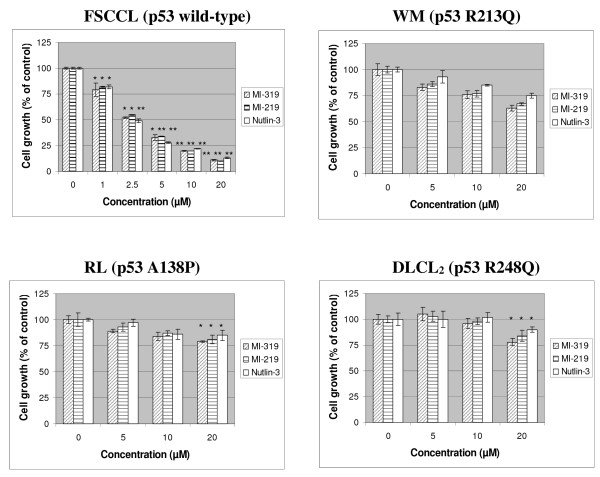
**Effect of MI-319, MI-219 and Nutlin-3 on cell proliferation *in vitro***. Established tumor cell lines. Cells were grown for 48 hours. The number of viable cells was determined by MTT assay.

Cell growth was examined by MTT assay [26]. As shown in Fig. 2, MI-319, MI-219 and Nutlin-3 demonstrated similar potency and all effectively inhibited the growth of FSCCL in a dose-dependent manner. For a 48-hour exposure, the concentration that leads to 50% inhibition of proliferation (IC50) of FSCCL is estimated to be 2.5 μM for all three compounds. For the three cells with mutant p53, IC50 can not be determined yet with drug concentrations up to 20 μM. Therefore, the three compounds exhibited approximately 10-fold selectivity in cells with wild-type p53 over cells with mutant *p53*. Interestingly, the three cells with mutant p53 responded differentially to all the three compounds. WM exhibited the strongest response, whereas DLCL_2 _exhibited the weakest response (Fig. 2).

**Figure 3:**
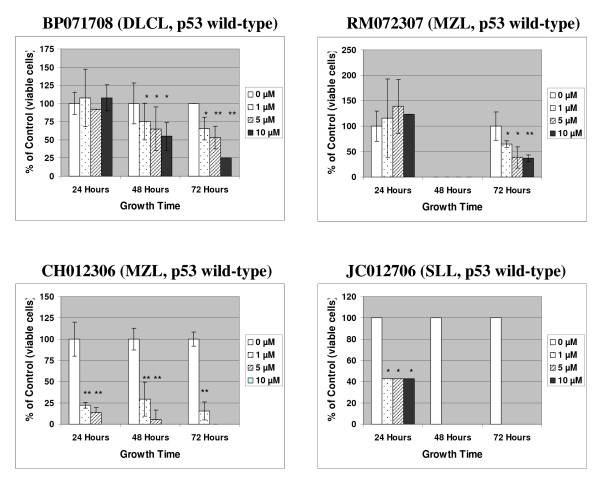
**Effect of MI-319, MI-219 and Nutlin-3 on cell proliferation *in vitro***. Mononuclear cells isolated from lymphoma patients - BP071708 is diffuse large B-cell lymphoma (DLCL), RM072307 is marginal zone B-cell lymphoma (MZL), JC012706 is another marginal zone B-cell lymphoma (MZL), and CH012306 is small lymphocytic lymphoma (SLL). The number of viable cells was determined by trypan blue exclusion test. Data represents mean of three independent experiments. * represents p < 0.05 ** represents p < 0.01.

In order to get a sense of clinical relevance of MI-319, we isolated, cultured and treated mononuclear cells from four B-cell lymphoma patients. As shown in Fig. 3, MI-319 showed significant cytotoxic effect on all four primary cultures.

### FSCCL cells exhibited increased protein levels of p53, MDM2, p21 and cleaved PARP after treatment with MI-319, MI-219 or Nutlin-3

**Figure 4:**
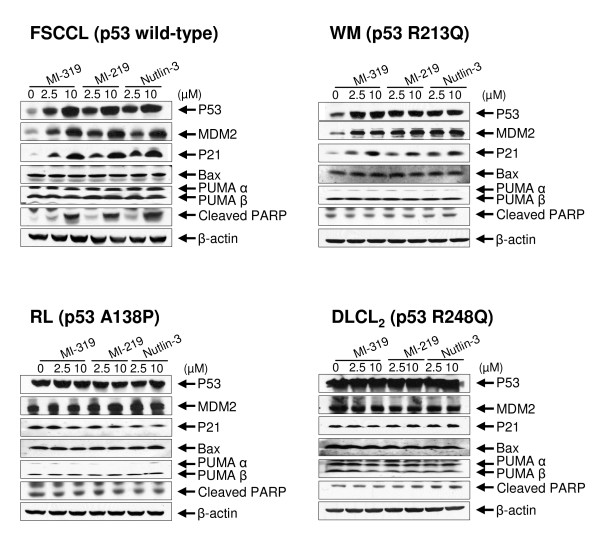
**Gene expression profiling by Western blotting**. Cells were treated with indicated chemical or equal volume of 100% DMSO for 12 hours.

Treatment with MI-319, MI-219 or Nutlin-3 for 12 hours led to similar increase in protein levels of p53, MDM2, p21 and cleaved PARP in FSCCL cells in a dose-dependent manner (Fig. 4). The levels of Bax and PUMA, however, were not affected by the treatments. In WM cells, which have R213Q p53 mutation, MI-319, MI-219 and Nutlin-3 induced increased protein levels of p53, MDM2 and p21, but not that of Bax, PUMA and cleaved PARP (Fig. 4). In RL and DLCL_2 _cells, treatment with the three compounds did not induce any significant protein level changes among the genes tested (Fig. 4).

### MI-319 induced differential cell cycle arrest responses among FSCCL, WM, RL and DLCL_2 _cells

**Figure 5:**
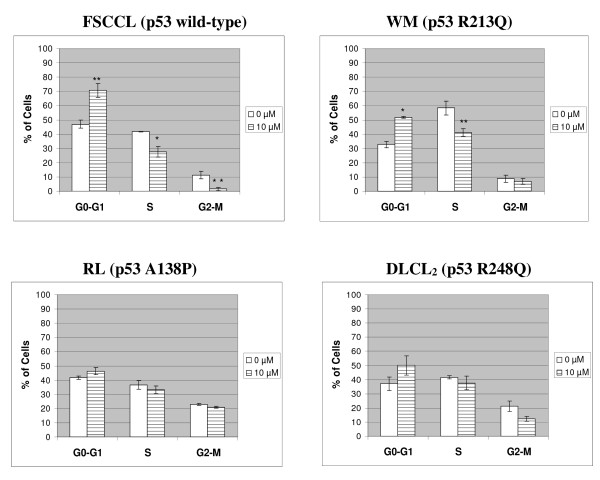
**Cell cycle analysis**. Cells were treated with MI-319 or equal volume of 100% DMSO for 24 hours. Numbers plotted here are the average of at least three independent experiments. * represents p < 0.05 and ** represents p < 0.01.

As shown in Fig. 5, treating FSCCL cells with 10 μM of MI-319 for 24 hours increased G0-G1 cells by approximately 24% and decreased S and G2-M cells by approximately 14% and 10%, respectively. In comparison, WM, RL and DLCL_2 _cells exhibited weaker and differential responses. In WM, G0-G1 cells increased by approximately 19%, while S and G2-M cells decreased by approximately 17% and 2%, respectively (Fig. 5). In RL, G0-G1 cells increased by approximately 5%, while S and G2-M cells decreased by approximately 3% and 2%, respectively (Fig. 5). In DLCL2, G0-G1 cells increased by approximately 13%, while S and G2-M cells decreased by approximately 4% and 9%, respectively (Fig. 5).

### MI-319 induces apoptotic cell death only in FSCCL cells

**Figure 6:**
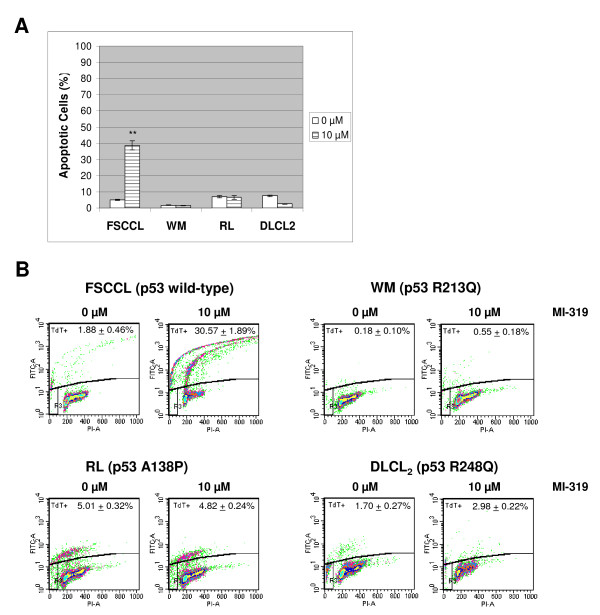
**MI-319 induces cell apoptosis in FSCCL cells only**. (A) Cells were stained with Annexin V-FITC and quantification of the percentage of apoptotic cells was done with a Coulter EPICS 753 flow cytometer. (B) Tunnel assay. TdT+ cells (apoptotic cells) were assessed with a Coulter EPICS 753 flow cytometer. Numbers plotted here are the average of three independent experiments. * represents p < 0.05 and ** represents p < 0.01.

Although the protein levels of pro-apoptotic p53 target genes such as Bax and PUMA are not affected by treatment with MI-319 (Fig. 4), in FSCCL cells however, there was a dose-dependent increase in cleaved PARP, which is one of the hallmarks of apoptotic cell death. To further investigate whether apoptotic mechanisms were involved, we analyzed cell death by Annexin V staining and Tunnel assay. As shown in Fig. 6, both experiments revealed that treating cells with 10 μM of MI-319 for 24 hours induced significant apoptotic cell death only in FSCCL cells.

### MI-319 has significant anti-lymphoma activity in FSCCL systemic SCID mouse model

**Figure 7:**
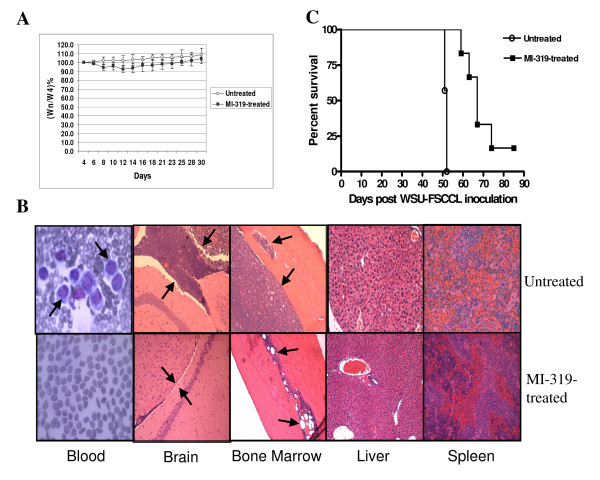
**Survival of FSCCL-SCID mice is prolonged with the treatment of MI-319**. (A) Treatment with MI-319 did not result in significant body weight changes (> 15%). Wn=mouse body weight at day (n); W4 = mouse body weight at day (4) when the administration of MI-319 was started. (B) H&E staining of mouse blood smear and sections of mouse brain, bone marrow, liver, and spleen. Arrows in the upper blood panel (untreated) point to lymphocytes, indicating lymphoma involvement. Lower brain panel (MI-319-treated) is an example of no involvement by lymphoma. Note the thin meningeal lining of the brain (between opposing arrows). In comparison, the upper panel (untreated) shows examples of involvement of the meninges by lymphoma. Note the expanded space between the opposing arrows indicative of lymphoma cell infiltration. For the bone marrow (femurs) sections, lower panel (MI-319-treated) shows examples of normal marrow and upper panel (untreated) shows examples of involvement by WSU-FSCCL (note the cavities filled with lymphocytes). (C) The survival percentage of untreated and MI-319-treated mice is plotted against days post FSCCL inoculation. The control animals have a median survival day of 51.6, whereas the treatment animals have a median survival day of over 66.0. n = 7 for control group, n = 6 for treatment group, p < 0.0001.

MI-319 was administered orally to the animals at 300 mg/kg twice a day (BID) for 7 days. The dose schedule was adopted from a previous study with MI-219 [8]. At this dose, MI-319 displayed no major adverse effect (> 15%) on body weight gain by the treated animals (Fig. 7A), suggesting it was tolerated well. After the administration of MI-319 was stopped, all animals gained weight (Fig. 7A). At day 51, when first control mouse died, one mouse from the treatment group was sacrificed and tissue samples were harvested. The effectiveness of MI-319 was first demonstrated by pathological examination of stained mouse tissues. As shown in Fig. 7B, blood, brain and bone marrow from the control mouse showed heavy involvement by lymphoma cells. In contrast, MI-319-treated mouse showed normal peripheral blood, brain and bone marrow with no apparent lymphoma involvement (Fig. 7B). Spleen and liver sections were also examined and neither control nor MI-319-treated mouse showed lymphoma involvement (Fig. 7B). Between days 51 and 52 post tumor cell inoculation, all seven control mice succumbed to the disease, with a median survival of 51.6 days (Fig. 7C). By contrast, until up to day 70 post tumor cell inoculation, only five out of seven MI-319-treated mice died, indicating a median survival of over 66.0 days. Thus, treatment with MI-319 conferred a > 28% (% ILS, 14.4 days) increase in median survival days and has statistically significant therapeutic impact (p < 0.0001). For the one remaining treated mouse, it remained alive after 90 days and appeared to achieve a complete cure.

## Discussion

Follicular lymphoma is the second most common type of NHL, which has increased incidence over the past three decades and is now the fifth most common cancer in the United States [1]. Current therapeutic tools for follicular lymphoma, such as monoclonal antibodies, radio-immunotherapy, vaccines and chemotherapeutic agents, all have limitations [27]. In an attempt to search for a targeted and less toxic agent that can be administered orally, we evaluated the anti-lymphoma activity of MI-319 in a follicular small cleaved cell lymphoma cell line established in our laboratory. Data obtained in our studies is encouraging and is consistent with the following statements: i) MI-319 is able to bind to MDM2 protein with a high affinity that is over 500-fold more potent than a natural p53 peptide; ii) MI-319 effectively inhibited proliferation of FSCCL cell (p53 wild-type) *in vitro*, with IC50 value of 2.5 μM for 48-hour treatment; iii) Inhibition of FSCCL cell proliferation by MI-319 involves induction of both cell cycle arrest and apoptotic death; iv) MI-319 displayed potent anti-tumor efficacy in the FSCCL-SCID mouse model.

MI-319 was designed to stabilize p53 protein in cells by blocking the MDM2-p53 interaction. Although many genes in addition to p53 are usually altered in tumors, recent studies suggest that restoring p53 function alone is sufficient to cause regression of established sarcomas, lymphomas, and liver tumors in mice [28-30]. Therefore, restoring functional p53 activity by using small molecules, such as MI-319, to block MDM2-p53 interaction and stabilize p53 protein is an attractive pharmacological approach. Since the discovery of the Nutlins [6], there has been a great deal of interest in the evaluation of small-molecule inhibitors of the MDM2-p53 interaction against various types of cancer [9]. Currently there are two major classes of such small-molecule inhibitors. One class is represented by Nutlin-3 [6,31,32]; the other one is represented by MI-219 [8]. MI-319 is a very close analogue of MI-219. In our fluorescence polarization-based competitive binding assay, MI-319 exhibited a binding affinity to human MDM2 protein that is slightly higher than that of MI-219 and Nutlin-3. Nevertheless, the three compounds have similar potency against the cells tested in this report in terms of growth inhibition and regulation of expression of p53 target genes, such as MDM2, p21, Bax and PUMA. Therefore, we believe that MI-319, MI-219 and Nutlin-3 are probably equal as an MDM2 antagonist. In our remaining cell cycle analysis, apoptotic cell death assays and animal model studies, we assessed only MI-319 simply due to availability issues.

**Figure 8:**
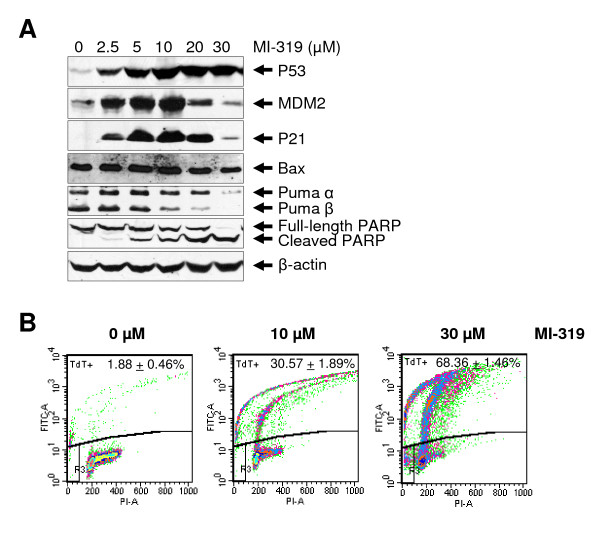
**MI-319-induced FSCCL apoptotic cell death might be p53 transcription-independent**. (A) FSCCL cells were treated for 12 hours and Western blotting experiments were performed to examine the protein levels of p53, MDM2, p21, Bax, PUMA and PARP. (B) FSCCL cells were grown for 24 hours after drug treatment and apoptotic cell death was assessed with Tunnel assay.

One of the mechanisms of MI-219- or Nutlin-3-induced apoptosis is thought to occur via p53's transcriptional program by up-regulating the expression of pro-apoptotic p53 target genes such as Bax and PUMA [8,33,34]. In our studies, we observed that exposing lymphoma cells to MI-319 for 24 hours with concentrations up to 10 μM induced significant apoptotic cell deaths only in FSCCL, which bears wild-type p53. However, protein levels of neither Bax nor PUMA were up-regulated by MI-319, MI-219, or Nutlin-3. We treated FSCCL cells for 24 hours with higher drug concentrations of 20 and 30 μM and found that MI-319-induced apoptosis was apparently p53 transcription-independent. At MI-319 concentrations of 20 and 30 μM, FSCCL exhibited decreased proteins levels of p53 transcriptional target genes, such as MDM2, p21, Bax and PUMA. However, cleavage of PARP and the number of TdT-positive cells (apoptotic cells) increased significantly in a dose-dependent manner (Fig. 8). It has been reported by Vaseva et al. that the transcription-independent mitochondrial p53 program is a major contributor to Nutlin-induced apoptosis in tumor cells [35]. It appears that Nutlin-induced mitochondrial p53 translocation is rapid and associated with cytochrome *C *protein release that precedes induction of p53 target genes [35]. Furthermore, blocking the transcriptional arm of p53 not only fails to inhibit, but greatly potentiates Nutlin-induced apoptosis [35]. We speculate that MI-319-induced apoptosis of FSCCL cells may also occur mainly via the transcriptional-independent mitochondrial p53 program.

In our studies, we assessed FSCCL side by side with three other cells that have mutant p53-WSU-WM (R213Q), RL (A138P) and WSU-DLCL_2 _(R248Q). Interestingly, the cells expressing the three mutants behaved differently in terms of cell proliferation, cell cycle arrest and expression of some of p53's target genes, such as MDM2 and p21. WM responded the strongest among the three and RL and DLCL2 responded much weaker. Previous reports have documented that these three p53 mutants still retain some of wild-type p53 protein's regulatory functions. When A138P and R248Q mutants were expressed in p53 null cells, it was found that both of them still retain a little (< 5%) of p53's regulatory activities [36,37]; according to the studies by Pan et al., the R213Q mutant is still partially functional [38] and therefore probably retains much more of wild-type p53's regulatory activities. Compared with wild-type p53, R213Q mutant p53 protein has a weaker transactivating activity for p21 gene [38]. Our gene expression profiling data obtained by Western blotting agreed quite well with this result. Treatment with MI-319, MI-219 or Nutlin-3 all led to dose-dependent up-regulation of p21 protein in WM cells, but appeared less robust than that in FSCCL. Overall, it appeared that the different responses of the three cells with p53 mutants correlated very well with the level of wild-type p53's regulatory activities retained by the corresponding p53 mutant proteins.

The main goal of our study is to find a novel agent that holds promises to make its way into clinical trials for the treatment of follicular lymphoma. Thus, we tested the anti-lymphoma activity of MI-319 *in vivo *by using a systemic FSCCL SCID mouse model. MI-319 given orally for one week showed no major toxicity, such as > 15% weight loss in treated animals, whereas the treatment showed a significant therapeutic impact (p < 0.0001); conferring a more than 28% (14.4 days) increase in life span (ILS).

## Conclusion

Our studies showed that MI-319, MI-219 and Nutlin-3 have similar potency as an MDM2 antagonist. MI-319 has potent anti-lymphoma activities against FSCCL cells. It stabilizes p53 protein and induces cell cycle arrest and apoptosis in follicular lymphoma cells that retain wild-type *p53*. When administered orally to the animals, MI-319 showed significant anti-lymphoma activity. Our results provide confidence towards the development of MDM2 inhibitors for lymphoma patients in the clinic.

## Methods

### Chemical synthesis and competitive binding assay

MI-319 and MI-219 were synthesized by using methods published previously [39]. Nutlin-3 was purchased from Sigma-Aldrich. For cell culture experiments, MI-319, MI-219 and Nutlin-3 were dissolved in 100% DMSO to make 10 mM stock solutions which were kept at -70°C. Fluorescence polarization-based competitive binding assays were performed to determine the binding affinity of MI-319 and MI-219 with a recombinant His-tagged MDM2 protein. The assays were carried out as described previously [40].

### P53 genomic DNA and full-length cDNA sequencing

Genomic DNAs were extracted by adapting a procedure described previously [41]. The amount of genomic DNA was determined by UV absorption at 260 nm. 200 ng was used in each reaction of PCR amplification. Primers to amplify exons 5/6, 7, and 8/9 of human p53 and adjacent intronic sequences were adopted from the literature [24] with modification of the p53-E5/6-F primer sequence as 5'-ggaggtgcttacgcatgtttg-3'. Amplified PCR products were analyzed by agarose gel electrophoresis, cleaned with Wizard SV Gel/PCR Cleanup kit (Promega, Madison, WI), and sequenced directly. Sequencing was done with the Applied Biosystems ABI Prism 3700 sequencer (Applied Biosystems, CA). Sequencing reactions were performed using the ABI BigDye^®^Terminator v3.1.

In order to sequence full-length p53 cDNA, total RNAs were isolated from cells using the RNeasy^® ^Isolation Kit (Qiagen, Valencia, CA). The amount of total RNA was estimated by UV absorption at 260 nm. The extracted RNAs (2 μg of each sample) were reverse-transcribed with the ImProm-II(TM) Reverse Transcription System, following the manufacturer's instructions (Promega, Madison, WI). PCR reactions were performed subsequently to amply p53 cDNA with an Eppendorf AG Mastercycler (Hamburg, Germany). Two pairs of primers were used to amplify the full-length p53 coding cDNA sequence. The sequence of the primers is as follows: p53-F1/p53-R1 (5'-aagtctagagccaccgtcca-3'/5'-catagggcaccaccacacta-3'), p53-F2/p53-R2 (5'-gtggaaggaaatttgcgtgt-3'/5'-gtggggaacaagaagtggag-3'). The PCR products were analyzed by agarose gel electrophoresis, cleaned with Wizard SV Gel/PCR Cleanup kit (Promega, Madison, WI), and sequenced directly.

### Cell culture

Cell lines FSCCL, WM and DLCL_2 _were established in our laboratory [25,42,43]. The cell line RL was purchased from the American Type Culture Collection (ATCC, Rockville, MD, USA). Mononuclear cells were isolated from four lymphoma patients - BP071708 is diffuse large B-cell lymphoma (DLCL) intermediate grade; RM072307 is marginal zone B-cell lymphoma (MZL) low grade; JC012706 is another marginal zone B-cell lymphoma (MZL) low grade; and CH012306 is small lymphocytic lymphoma (SLL) low grade. All patients had stage IV disease. Lymphoma cells were isolated by Ficoll gradient centrifugation (GE Healthcare, Little Chalfont, United Kingdom), seeded into growth medium right away or aliquoted into fetal bovine serum with 10% DMSO and cryopreserved in liquid nitrogen. Studies involving human tissues were done according to IRB-approved protocol and all patients had signed informed consent prior to tissue procurement. Cells were usually seeded at a density of 2 × 10^5 ^viable cells per ml in 24-well or 6-well culture plates (Costar, Cambridge, MA), or 10-cm cell culture dishes (Corning Inc., Corning, NY). All cells were maintained in RPMI 1640 medium supplemented with 10% fetal bovine serum (Hyclone Laboratories, Logan, Utah) and 1% Penicillin-Streptomycin (Invitrogen, Carlsbad, CA), at 37°C in a humidified incubator with 5% CO_2_. The number of viable cells was determined by trypan blue exclusion test with trypan blue (0.4%) purchased from Sigma Chemical Co. (St. Louis, MO) and MTT assay [26]. Statistical analysis was done using the *t *test (two tailed) with 95% confidence intervals between treated and untreated samples. P < 0.05 was used to indicate statistical significance.

### Western blot

Cells were collected by centrifugation, washed twice with cold PBS, and lysed at 4°C in lysis buffer containing protease inhibitors as described previously [44]. Total protein content in lysates was estimated by the Bradford method [45]. The primary antibodies used in the experiments included p53 (Cell Signaling, Danvers, MA), MDM2 (R&D System, Minneapolis, MN), p21 (Cell Signaling, Danvers, MA), Bax (Sigma, St. Louis, MO), PUMA (Sigma, St. Louis, MO), PARP (Cell Signaling), and β-actin (Sigma).

### Cell cycle analysis

Cells were collected by centrifugation and washed twice with cold PBS. Cell pellets were resuspended in 0.5 ml of cold PBS and fixed in 4.5 ml of 70% ethanol and stored at 4°C. On the day of analysis, cells were collected by centrifugation and each pellet was resuspended in 1 ml of staining buffer, which contains 50 μg/mL of propidium iodide, 100 μg/mL of RNase A, and 0.1% of Triton X-100. The cell suspensions were incubated in the dark for 30 minutes at room temperature and subsequently analyzed on a Coulter EPICS 753 flow cytometer for DNA content. The percentage of cells in different phases of the cell cycle was determined using a ModFit 5.2 computer program.

### Apoptosis analysis

Apoptotic cell death was determined with two methods: Annexin V-FITC staining and Tunnel assay. Annexin V-FITC staining kit and ApoDIRECT In Situ DNA Fragmentation Assay (Tunnel assay) kit were purchased from BioVision (Mountain View, CA). Experiments were performed by following the manufacturer's instructions. Quantifications of the percentage of apoptotic cells were done with a Coulter EPICS 753 flow cytometer.

### FSCCL systemic xenograft model

All animal studies were conducted according to Animal Investigation Committee (AIC)-approved protocol of Wayne Sate University. This systemic model was initiated by injecting 20 × 10^6 ^FSCCL cells via the tail vein (iv) of acclimated 3-4 week old female severe combined immune deficient mice (ICR-SCID) (Taconic Farms, Germantown, NY). Animals were monitored daily for changes in weight, side effects of the treatment or signs of any sickness. 8-10 weeks post inoculation, symptoms such as diarrhea, dehydration, ascites, lethargy, paralysis and/or general weakness became evident, thus animals were euthanized, tissues such as liver, spleen, bone marrow, lymph nodes, blood and brain were harvested and subjected to H&E staining to evaluate pattern of dissemination, involvement and confirmation of engraftment. Engraftment rate for this model is 100%.

### Animal preclinical efficacy trial design

The *in vivo *anti-tumor activity of MI-319 was assessed against our FSCCL xenograft model. To ensure randomness, 14 animals were combined in a single cage and inoculated with FSCCL. Seventy-two hours later, mice were pooled and 2 groups of seven animals each were randomly and unselectively assigned to two interventions; control and MI-319-treated group. MI-319 was administered orally at 300 mg/kg BIDx7.

### Percent Increase in Host life Span (%ILS)

%ILS was calculated by subtracting the median day of death of the treated tumor-bearing mice from median day of death of the tumor-bearing control divided by the median day of death of the tumor-bearing control animals. Statistical analysis of data was carried out with GraphPad Prism software. Survival distribution of the treated (T) and control (C) groups was compared using the log-rank test. In this report, survival was characterized as the duration of the animal's life span 24-hours after the initiation of the xenograft until an observed event (euthanasia or death).

### Pathological evaluation of mouse tissues

Necropsy was carried out to determine extent of macroscopic lymphoma. Major organs including the brain, femur (for bone marrow), liver, and spleen were harvested for microscopic examination. In addition, peripheral blood smears were examined for evidence of circulating lymphoma cells.

## Competing interests

Ascenta Therapeutics has licensed the technology related to MI-319 and its analogues from the University of Michigan. S. Wang owns stocks and stock options in Ascenta Therapeutics and serves as a consultant and its scientific advisor. The University of Michigan also owns stocks in Ascenta Therapeutics. D. Yang is one of the co-founders of Ascenta. He owns stocks and stock options in Ascenta and serves as the Senior Vice President of Research of the company.

## Authors' contributions

JW performed experiments and prepared the manuscript. AA, AS, SW, ASA, and ZN performed experiments. DY and SW synthesized MI-319 and MI-219. AMA-K provided patient samples and supervised the project. RMM designed the experiments, supervised the project and prepared the manuscript. All authors read and approved the final manuscript.
